# 100 years *Cell and Tissue Research*: the founders and their successors

**DOI:** 10.1007/s00441-025-03950-w

**Published:** 2025-01-27

**Authors:** Klaus Unsicker

**Affiliations:** Institute of Anatomy and Cell Biology, Department of Molecular Embryology, University of Freiburg, 79104 Freiburg, Germany

**Keywords:** CTR, Springer/Nature journals

## Abstract

One hundred years ago, *Cell and Tissue Research* was founded under the title “Zeitschrift für Zellen- und Gewebelehre,” later “Zeitschrift für Zellforschung und mikroskopische Anatomie.” The founders were four eminent German and Swiss cell biologists and zoologists, R. Goldschmidt, W. von Möllendorff, H. Bauer, and J. Seiler.

One hundred years ago, *Cell and Tissue Research* was founded under the title “Zeitschrift für Zellen- und Gewebelehre,” later “Zeitschrift für Zellforschung und mikroskopische Anatomie.” The founders were four eminent German and Swiss cell biologists and zoologists, R. Goldschmidt, W. von Möllendorff, H. Bauer, and J. Seiler.

Richard Baruch-Benedikt Goldschmidt was born in Frankfurt/Main (1878). He studied Medicine and Zoology with Otto Bütschli and Carl Gegenbaur at the University of Heidelberg and did his PhD and habilitation (private docentship) with Richard Hertwig at Munich University. In 1914, he was appointed to the Kaiser-Wilhelm-Institute (now Max-Planck-Institute) of Biology in Berlin. Dismissed by the Nazi government in 1935 because of his Jewish descent, he migrated to the USA, where he became professor of Genetics and Cell Biology at UC Berkeley. In 1947, he became a member of the National Academy of Sciences. Among Goldschmidt’s numerous discoveries are the constant cell number in the nematode *Caenorhabditis*, the development and recovery of stress expression in *Drosophila*, and the idea that genes are macromolecules (although he thought they were proteins).

Wilhelm von Möllendorff was born in 1887 in Manila, Philippines, where his father managed the German Consulate. He attended High Schools in Tilsit (now Russian territory Kaliningrad) and Frankfurt/Main and studied Archeology and Medicine at Heidelberg University. He then started a scientific career in Anatomy at the University of Greifswald, where he did a habilitation. He then was appointed extraordinary professor at Freiburg University and subsequently full professor at Hamburg and Kiel universities. In 1927, he returned to Freiburg University.

In December 1932, von Möllendorff was elected as rector of Freiburg University for the academic year 1933/34 but stepped back from office after a few days, since he refused to obey the order of the Nazi government and dismiss Jewish colleagues. Two of von Möllendorff’s collaborators during this period were Wolfgang Bargmann and Hans Adolf Krebs. Bargmann moved with him to the Anatomy Department of Zurich University in 1935 and, after World War II, became editor of CTR. Krebs, born into a Jewish family, left Germany, went to England, and received the Nobel Prize for Physiology or Medicine (“Krebs cycle”). Wilhelm von Möllendorff never returned to Germany; he died in 1944 during a skitour in Lenzerheide, Switzerland. Von Möllendorff’s most prominent discoveries are in the fields of developmental and cell biology, vital dye staining, kidney architecture and function, mitotic cell division, and cancer.

Hans Bauer (Bauer 1936), born 1904 in Hamburg, received his PhD for work he had done in Alfred Kühn’s lab in Göttingen. He made important discoveries in chromosome research, particularly on giant chromosomes in Drosophila (Beermann 1988), and became director of several Biology Institutes of the Kaiser-Wilhelm-Gesellschaft, later Max-Planck-Gesellschaft, in Berlin-Dahlem, Wilhelmshaven, and Tübingen. He died in 1988.

Jakob Seiler, born 1886 in Merishausen, Switzerland, was a Swiss biologist, who studied Zoology at the universities of Geneva and Zurich. His speciality in science was intersexuality in invertebrates. He received an extraordinary professorship in cell biology and genetics from the University of Munich in 1927 and was a professor at the ETH Zurich from 1933 to 1957. He became a member of the German Academy Leopoldina in 1940 and the Bavarian Academy of Science in 1952 and received the Swiss Prize in science “Marcel Benoist.”

Starting with volume 2 (1925), the journal was edited by R. Goldschmidt and W. von Möllendorff. From 1949 to 1960, W. Bargmann and J. Seiler were the editors. Wolfgang Bargmann continued to be editor until 1978, before handing over to Andreas Oksche. Klaus Unsicker was the editor from 1996 to 2023; the present editors are David Kelsell and Vera Kozjak-Pavlovic.

Numerous “cooperating editors” were involved in editing the journal over the years. There are too many to list them here, but it is important to remind us all the time that the quality of a journal is the result of a strong collaborative effort of many excellent scientists, not to forget those who provide the support in the offices.

I wish CTR many more years of excellence!

Klaus Unsicker.

## Sources and website links

“https://de.wikipedia.org/w/index.php?title=Richard_Goldschmidt&oldid=235505789”.

Stefan Schulz: Möllendorff, Wilhelm von (https://hls-dhs-dss.ch/de/articles/014554 In: Historisches Lexikon der Schweiz.

Christian Baertschi: Jakob Seiler. (https://his-dhs-dss.ch/de/articles/044459) In: Historisches Lexikon der Schweiz.

## Data Availability

No datasets were generated or analysed during the current study.
